# Optimization of the Alizarin Red S Assay by Enhancing Mineralization of Osteoblasts

**DOI:** 10.3390/ijms24010723

**Published:** 2022-12-31

**Authors:** Aline Bernar, Jennifer Viktoria Gebetsberger, Monika Bauer, Werner Streif, Michael Schirmer

**Affiliations:** 1 Department of Pediatrics I, Medical University of Innsbruck, 6020 Innsbruck, Austria; 2 Department of Internal Medicine, Clinic II, Medical University of Innsbruck, 6020 Innsbruck, Austria

**Keywords:** alizarin red S assay, mineralization, bone, osteoblasts, calcium chloride, calcitonin

## Abstract

The alizarin red S assay is considered the gold standard for quantification of osteoblast mineralization and is thus widely used among scientists. However, there are several restrictions to this method, e.g., moderate sensitivity makes it difficult to uncover slight but significant effects of potentially clinically relevant substances. Therefore, an adaptation of the staining method is appropriate and might be obtained by increasing the mineralization ability of osteoblasts. In this study, cell culture experiments with human (SaOs-2) and murine (MC3T3-E1) osteoblasts were performed under the addition of increasing concentrations of calcium chloride (1, 2.5, 5, and 10 mM) or calcitonin (1, 2.5, 5, and 10 nM). After three or four weeks, the mineralization matrix was stained with alizarin red S and the concentration was quantified photometrically. Only calcium chloride was able to significantly increase mineralization, and therefore enhanced the sensitivity of the alizarin red S staining in a dose-dependent manner in both osteoblastic cell lines as well as independent of the cell culture well surface area. This cost- and time-efficient optimization enables a more sensitive analysis of potentially clinically relevant substances in future bone research.

## 1. Introduction

Bone is a highly dynamic organ that exerts important functions in the bodies of air-breathing vertebrates, such as support and protection of soft tissues, locomotion, calcium and phosphate storage, or harboring of bone marrow [1]. Two processes are important for the development and maintenance of the skeletal system: bone remodeling and modeling. Growth and mechanically induced adaption of bone depends on bone modeling, while bone remodeling is responsible for removal and repair of damaged bone. They are essential for maintaining the integrity of the adult skeleton and mineral homeostasis [2]. Bone (re)modeling includes two important cell types: Osteoclasts, which are responsible for bone resorption, and osteoblasts, which conduct bone formation [1,3]. Osteoblasts arise from pluripotent mesenchymal stem cells and account for 4–6% of total resident cells in the bone. Differentiation into osteoblasts requires the expression of specific genes as well as the synthesis of bone morphogenetic proteins and members of the wingless pathway [4]. Mature osteoblasts are essential for the deposition of matrix followed by its mineralization [4]. As matrix mineralization represents the last step of differentiation, it is a very important outcome measure for determining the differentiation efficacy of osteoblasts in bone research [5,6].

Numerous techniques are currently used to characterize biological mineralization in intact tissues and cell culture systems, including fluorescent dyes such as xylenol orange, calcein blue [7], IRDye 800CW BoneTag, and OsteoSense 800 [6]. Additionally, alkaline phosphatase (ALP) enzymatic activity can be assessed [8]. However, in vitro mineralization—and to be more precise, the presence of calcium phosphate (hydroxyapatite) in osteoblastic cell cultures—is typically assessed by von Kossa and alizarin red S staining [9]. In von Kossa stains, silver ions react with the anions (phosphates, sulfates, or carbonates) of calcium, forming dark brown or black precipitates, which can be observed with the naked eye [10,11]. Alizarin red S is considered the gold standard for the detection and quantification of mineralization since this anthraquinone derivative reacts more specifically with calcium cations through a chelation process. An alizarin red S-calcium complex is formed thereby, resulting in a bright red stain that can also be observed visually [9]. This method consists of the fixation of the cells with formalin, followed by staining with alizarin red S dye and examining the staining under a phase-contrast microscope. For quantitative analysis, the dye can be extracted and quantified by a colorimetric assay [5,12].

However, this method has several drawbacks and restrictions. In particular, alizarin red S assay demonstrates only moderate sensitivity, which makes early differentiation or slight but significant differences in mineralization and osteoblast behavior difficult to detect [5]. Therefore, an optimization of the alizarin red S assay is appropriate; the aim of this article was to achieve this by enhancing the mineralization capacity of osteoblasts.

Naturally, bone resorption induces a local increase in extracellular calcium concentrations within the immediate vicinity of osteoclasts, which provide pre-osteoblasts with a signal that modulates their subsequent responses, such as migration and proliferation [13]. A calcium-sensing receptor is thus essential in calcium homeostasis and has been shown to be present on osteoblastic cell lines such as murine MC3T3-E1 [13] and human SaOs-2 [14]. By stimulating this receptor with extracellular calcium, chemotaxis and DNA synthesis could be induced experimentally in tissue culture models [13,15]. Additionally, Yamauchi et al. were able to show that increasing concentrations of extracellular calcium chloride (2.8 mM and 3.8 mM) or of the calcium receptor agonist NPS-R467 (1 and 3 µM) are able to regulate differentiation and thus to enhance mineralization of MC3T3-E1 osteoblasts [16]. Similar experiments with fetal rat calvarial cells (FRCs) showed that increased mineralization in the presence of extracellular calcium (1.8 and 2.5 mM) is not simply due to the increased bioavailability of calcium ions, but rather the result of an increase in both proliferation and maturation of osteoblasts [17].

In addition to calcium, the 32-amino-acid peptide hormone calcitonin—which is secreted from the parafollicular cells of the thyroid gland in humans and other chordates [18]—is also important for the maintenance of bone homeostasis [1]. It has been well documented that calcitonin is able to inhibit osteoclastic bone resorption, however, its role in osteoblastic bone formation remains controversial, since either stimulating, inhibitory, or no (direct) effects on osteoblasts have been reported [18,19]. As such, osteoblasts isolated from the calvariae of 1-day-old newborn Sprague–Dawley (SD) rats and cultured in 3 nM calcitonin-conditioned medium were able to increase their mineralization ability [20]. This conditioned medium was derived from calcitonin-treated osteoclasts, however, and thus it was hypothesized that the effect might be mediated indirectly by osteoclasts. Further experiments with bone marrow monocytes isolated from femoral and tibial bones of 8-week-old SD rats showed that calcitonin indeed increases the expression of Wnt10b, a clastokine able to increase osteoblast activity [20]. Weiss et al. showed that calcitonin has a stimulatory effect when administered to rats implanted with a demineralized bone matrix (prepared from rat diaphysis) during the initial phases of bone formation (day zero to seven) but is inhibitory when given in later stages of bone formation (day eight to fourteen) [21]. It is however difficult to interpret direct effects of calcitonin on bone formation, since osteoblasts obviously lack the expression of calcitonin receptors [22,23]. The observed stimulatory effects could thus either be explained by non-specific interactions of calcitonin with receptors different to already described ones [24] or—as mentioned before—by indirect interactions with osteoclasts [20,24].

So far, mineralization studies have been performed with or without addition of calcium chloride and calcitonin; examples are summarized in Table S1 in the Supplementary Material and discussed throughout the text. The main aim of this study was to increase the mineralization ability of osteoblasts in order to gain a better signal strength in the alizarin red S assay. An increase of the signal at baseline condition is beneficial, especially if inhibitory effects on osteoblasts exhibited by potentially clinically relevant substances are investigated. It has been described that various osteoblastic cell models (e.g., primary cells and osteoblasts induced from pluripotent stem cells, immortalized and malignant cell lines) show interspecies or genomic differences, inconsistent results regarding cell mineralization, and, e.g., do not mirror the whole range of phenotypic changes [25]. Thus, murine MC3T3-E1 and human SaOs-2 osteoblastic cell lines were used for the experiments. These two cell lines have been shown to form and mineralize a collagenous extracellular matrix, show a homogenous phenotype, and are unlimited in the number of cells [26,27]. Osteoblastic cells were stimulated with increasing concentrations of calcium chloride and calcitonin for three or four weeks, and subsequently alizarin red S staining assays were performed. To exclude any possible influence of the surface area available for osteoblasts to mineralize, experiments were performed in 96- and 24-well plates for comparison.

## 2. Results

### 2.1. Alizarin Red S Staining of Murine MC3T3-E1 Osteoblasts

**Figure 1:**
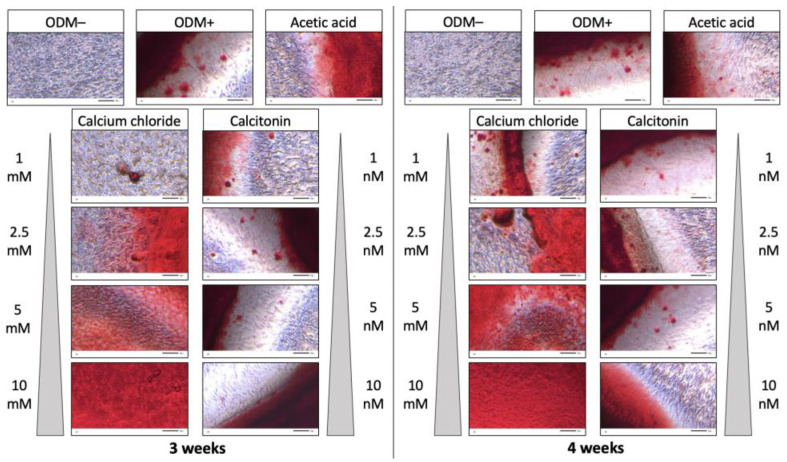
Alizarin red S staining of murine osteoblasts (MC3T3-E1) after incubation with calcium chloride or calcitonin. Cells were incubated with increasing calcium chloride (1, 2.5, 5, and 10 mM) or calcitonin (1, 2.5, 5, and 10 nM) concentrations for three (**left** panel) or four weeks (**right** panel) and subsequently stained with alizarin red S. Each condition was assessed in triplicate in three independent assays (n = 9). ODM− served as negative control (n = 6), ODM+ (n = 12) as reference, and acetic acid (n = 6) was used as an additional control as calcitonin was dissolved in acetic acid. Representative pictures of transmitted light microscopy at 20× magnification are shown. Scale bar is 50 µm. ODM; osteoblast differentiation medium.

Murine MC3T3-E1 osteoblasts were incubated with increasing concentrations of calcium chloride (1, 2.5, 5, and 10 mM) and calcitonin (1, 2.5, 5, and 10 nM). For the negative control, cells were grown only in α-minimum essential medium (αMEM) without supplementation with ascorbic acid and β-glycerophosphate (ODM−) so that no differentiation and thus mineralization was induced. The reference consisted of the common osteoblast differentiation medium (ODM+; αMEM medium with supplementation). Since calcitonin was dissolved in acetic acid, the effect of acetic acid alone was analyzed as well. Three independent experiments with triplicates except for the reference (n = 12) and the negative (n = 6) and acetic acid (n = 6) controls were performed. Representative pictures of the alizarin red S staining are shown in Figure 1.

As expected, cells incubated in ODM− medium did not show any mineralization compared to the ODM+ reference. Addition of acetic acid to differentiating osteoblasts did not show any effect on their mineralization behavior independent of time of incubation. Calcium chloride increased the mineralization of osteoblasts in a dose-dependent manner after three and four weeks. However, mineralization was obviously not enhanced when calcitonin was added to the osteoblast differentiation medium compared to the acetic acid control, independent of concentration and incubation time (Figure 1).

### 2.2. Quantification of Alizarin Red S Staining of Murine MC3T3-E1 Osteoblasts

**Figure 2:**
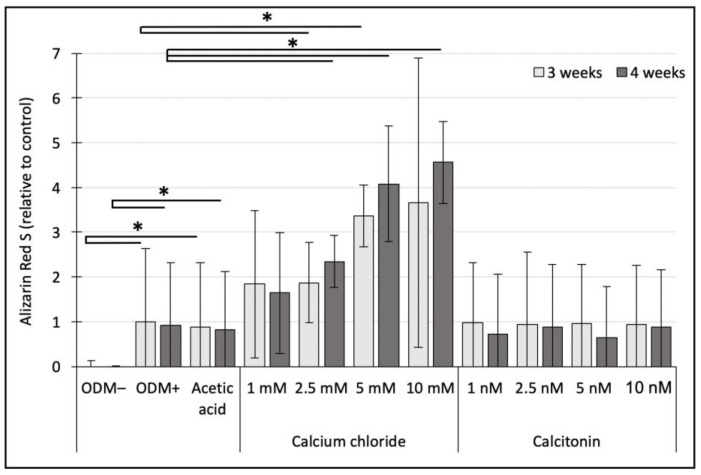
Photometric quantification of alizarin red S in murine MC3T3-E1 cells. Cells were incubated with increasing concentrations of calcium chloride (1, 2.5, 5, and 10 mM) or calcitonin (1, 2.5, 5, and 10 nM) for three or four weeks. Concentrations of alizarin red S are represented relative to the ODM+ reference after three weeks. Values represent means of three independent experiments in triplicate (n = 9) except for ODM+ reference (n = 12), the negative control (ODM−; n = 6) and the acetic acid control (n = 6) ± standard deviation. ODM; osteoblast differentiation medium. * Indicates a significant difference (*p* < 0.05).

After staining, the concentration of alizarin red S was measured photometrically. For quantification and better comparison, the ODM+ reference at three weeks was set as value 1. Results showed that there was no mineralization detectable in the ODM− negative control, with a significant difference compared to the ODM+ reference (three weeks, *p* < 0.001; four weeks, *p* < 0.001) and the acetic acid control (three weeks, *p* < 0.05; four weeks, *p* < 0.05), independent of incubation time (Figure 2). There was no significant difference between the ODM+ reference and the acetic acid control. After three weeks of incubation and relative to the ODM+ reference, a significant increase of solubilized calcium-bound alizarin red S concentrations could be detected when 2.5 mM (1.9-fold; *p* < 0.05) and 5 mM (3.4 fold; *p* < 0.001) calcium chloride was added to the osteoblast differentiation medium. Similar results were obtained when osteoblasts were incubated with calcium chloride for four weeks, with significant differences between the ODM+ reference and upon addition of concentrations as high as 2.5 mM (2.4-fold; *p* < 0.001), 5 mM (4.1-fold; *p* < 0.001), and 10 mM (4.6-fold; *p* < 0.001) (Figure 2).

No significant differences between the acetic acid control and osteoblasts incubated with increasing concentrations of calcitonin could be detected, independent of incubation time (Figure 2). Comparing the results of three and four weeks between the individual concentrations of calcium chloride or calcitonin, no significant differences could be detected (Figure 2).

### 2.3. Alizarin Red S Staining of Human SaOs-2 Osteoblasts

**Figure 3:**
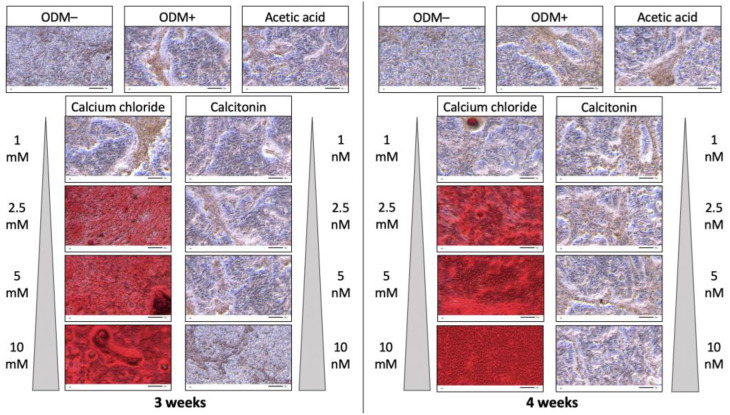
Alizarin red S staining of human osteoblasts (SaOs-2) after incubation with calcium chloride or calcitonin. Cells were incubated with increasing calcium chloride (1, 2.5, 5, and 10 mM) or calcitonin (1, 2.5, 5, and 10 nM) concentrations for three (**left** panel) and four weeks (**right** panel), and subsequently stained with alizarin red S. Each condition was assessed in triplicate in three independent experiments (n = 9). ODM− served as a negative control (n = 6), ODM+ (n = 12) as reference, and acetic acid (n = 6) was used as an additional control as calcitonin was dissolved in acetic acid. Representative pictures of transmitted light microscopy at 20x magnification are shown. Scale bar is 50 µm. ODM; osteoblast differentiation medium.

Microscopic images of alizarin red S-stained human SaOs-2 osteoblasts showed no mineralization in the ODM− control. However, there was also no visual increase of alizarin red S stains when osteoblasts were stimulated with ascorbic acid and β-glycerophosphate (ODM+), or upon addition of acetic acid, independent of incubation time (Figure 3).

However, a clear increase in mineralization was observed when calcium chloride was added to the osteoblast differentiation medium in a dose-dependent manner. This effect increased with a longer incubation time of four instead of three weeks (Figure 3). This outcome was not visible upon addition of calcitonin, independent of concentration and incubation time (Figure 3).

### 2.4. Quantification of Alizarin Red S Staining of Human SaOs-2 Osteoblasts

**Figure 4:**
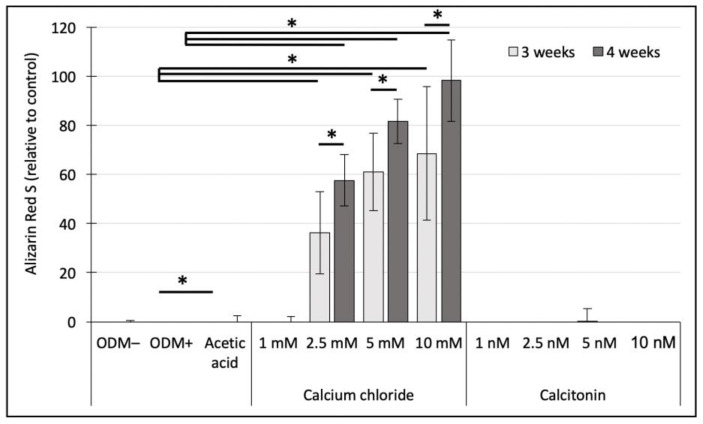
Photometric quantification of alizarin red S concentrations in human SaOs-2 cells. Cells were incubated with increasing concentrations of calcium chloride (1, 2.5, 5, and 10 mM) or calcitonin (1, 2.5, 5, and 10 nM) for three or four weeks. Concentrations of alizarin red S are represented relative to the ODM+ reference after three weeks. Values represent means of three independent assays in triplicate (n = 9) except for the ODM+ reference (n = 12), the negative control (ODM−; n = 6) and the acetic acid control (n = 6) ± standard deviation. ODM; osteoblast differentiation medium. * Indicates a significant difference (*p* < 0.05).

Quantification of alizarin red S staining in human SaOs-2 cells showed neither a detectable mineralization in the negative ODM− control, nor in the reference (ODM+) or acetic acid control, independent of incubation time (Figure 4). Mineralization of human SaOs-2 cells was only detectable when calcium chloride was added to the osteoblast differentiation medium (2.5–10 mM). Whereas no difference was obtained upon addition of 1 mM compared to the ODM+ reference, there was a significant increase in solubilized calcium-bound alizarin red S at calcium concentrations as high as 2.5 mM (36.3-fold; *p* < 0.001), 5 mM (61-fold; *p* < 0.001), and 10 mM (68.5-fold; *p* < 0.001) after three weeks of incubation. Similar differences between the ODM+ reference and upon addition of calcium chloride were observed after four weeks: at 2.5 mM by 57.6-fold (*p* < 0.001), at 5 mM by 81.6-fold (*p* < 0.001), and at 10 mM by 98.2-fold (*p* < 0.001) (Figure 4). Between the individual calcium chloride concentrations (2.5, 5, 10 mM), significant differences between three weeks and four weeks of incubation could be determined (2.5 mM, *p* = 0.006; 5 mM, *p* = 0.005; 10 mM, *p* = 0.015).

Addition of calcitonin did not show any significant increase in mineralization compared to the acetic acid control, independent of concentration and incubation time (Figure 4).

### 2.5. Influence of the Surface Area on the Mineralization of Murine MC3T3-E1 Osteoblasts

**Figure 5:**
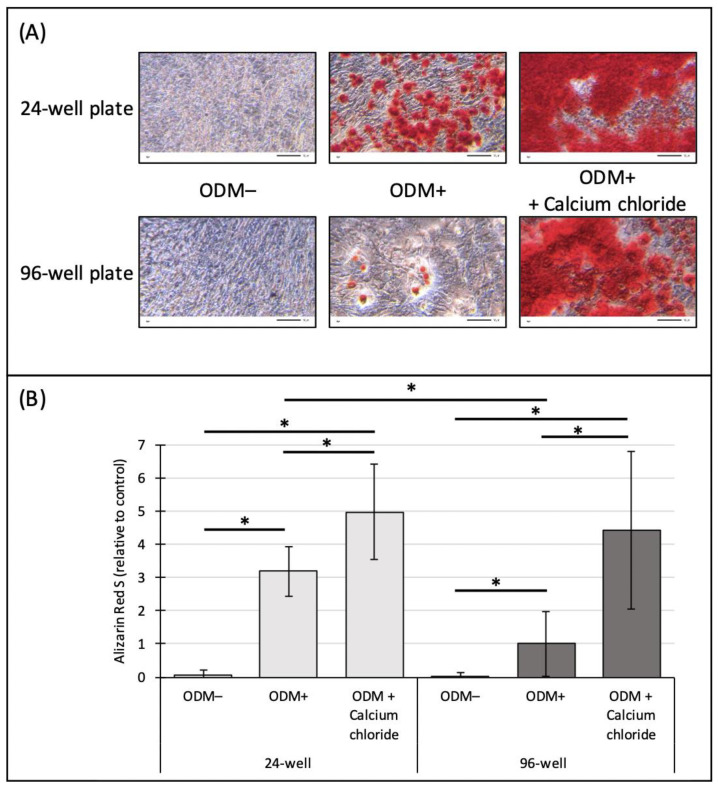
Effect of well size on alizarin red S staining of murine MC3T3-E1 osteoblasts. Murine osteoblasts were stimulated with osteoblast differentiation medium (ODM+) and in combination with calcium chloride (5 mM) in 24- or 96-well plates for three weeks before alizarin red S staining was performed. ODM− served as a negative control. (**A**) Representative pictures of alizarin red S-stained MC3T3-E1 osteoblasts assessed with transmitted light microscopy at 20× magnification are shown. Scale bar is 50 µm. (**B**) Concentrations of alizarin red S were calculated photometrically and are represented relative to the ODM+ reference of the 96-well plate. Means ± standard deviation from three independent experiments using sextuplicates are shown. * Indicates a significant difference (*p* < 0.001).

In order to investigate the mineralization capacity of osteoblasts depending on the available surface area and thus on size of the cell culture well, analyses were performed in parallel in 24-well and 96-well plates with murine MC3T3-E1 osteoblasts. Microscopic images showed that no mineralization was detectable in the ODM− control in neither the 24-well nor the 96-well plate (Figure 5A). Instead, mineralization was observed when osteoblasts were cultured in ODM+ medium in 24-well and 96-well plates, and a higher alizarin red S staining was already visible with the naked eye in 24-well plates (Figure 5A). There was a clear increase in mineralization of osteoblasts in both well sizes when 5 mM of calcium chloride was added to the osteoblast differentiation medium (Figure 5A).

For the photometric quantification of the alizarin red S staining, the ODM+ reference in the 96-well plate was set as value 1. Results showed that there was a significant difference between osteoblasts induced to mineralization (ODM+) compared to cells incubated only in αMEM medium (ODM−), independent of the size of the wells (24-well, *p* < 0.001; 96-well, *p* < 0.001) (Figure 5B). A significant difference, independent of the well size, was detected when 5 mM of calcium chloride was added compared to the negative control and the ODM+ reference (24-well/ODM−, *p* < 0.001; 24-well/ODM+, *p* < 0.001; 96-well/ODM−, *p* < 0.001; 96-well/ODM+, *p* < 0.001), confirming our previous results (Figure 2).

Comparing the mineralization capacity of osteoblasts cultured in 24- or 96-well plates, there was only a significant difference in alizarin red S concentrations at ODM+ conditions (24-well, 3.2-fold; 96-well, 1-fold; *p* < 0.001). No significant differences with ODM− or ODM+ supplemented with calcium chloride was observed between the 24- and 96-well plates (Figure 5B).

### 2.6. Influence of Surface Area on the Mineralization of Human SaOs-2 Osteoblasts

**Figure 6:**
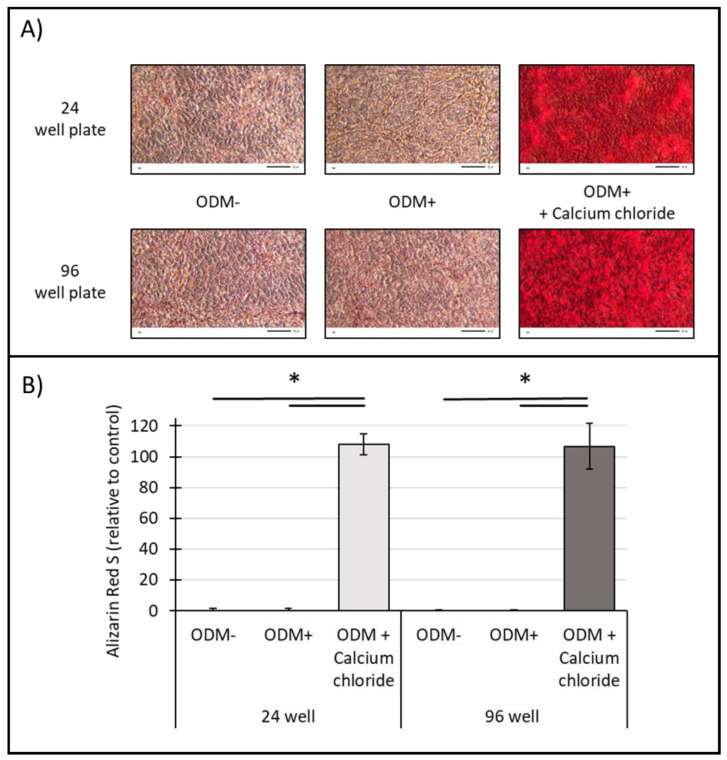
Effect of well size on alizarin red S staining of human SaOs-2 osteoblasts. Human osteoblasts were stimulated with osteoblast differentiation medium (ODM+) and in combination with calcium chloride (5 mM) in 24- or 96-well plates for three weeks before alizarin red S staining was performed. ODM− served as a negative control. (**A**) Representative pictures of alizarin red S-stained SaOs-2 osteoblasts assessed with transmitted light microscopy at 20x magnification are shown. Scale bar is 50 µm. (**B**) Concentrations of alizarin red S were calculated photometrically and are represented relative to the ODM+ reference of the 96-well plate. Means ± standard deviation from three independent experiments using sextuplicates are shown. * Indicates a significant difference (*p* < 0.001).

Analysis on the influence of available surface area on mineralization capacity was also performed for human SaOs-2 osteoblasts. Microscopic images showed no visible mineralization in the ODM− control, neither in the 24- nor in the 96-well plate (Figure 6A). Again, no mineralization was observed in the ODM+ reference (as already shown in Figure 3), independent of the size of the well. However, a strong alizarin red S staining and thus mineralization could be obtained when 5 mM calcium chloride was added to the osteoblast differentiation medium, without any visible difference between 24- and 96-well plates (Figure 6A).

Photometric quantification of the alizarin red S staining showed no significant difference between osteoblasts incubated in αMEM medium (ODM−) or in osteoblast differentiation medium (ODM+), independent of the well size (Figure 6B). A significant difference, independent of the available surface area, was only obtained when 5 mM of calcium chloride was added to differentiating osteoblasts, compared to the negative control (ODM−) and the ODM+ reference (24-well/ODM−, *p* < 0.001; 24-well/ODM+, *p* < 0.001; 96-well/ODM−, *p* < 0.001; 96-well/ODM+, *p* < 0.001).

Comparing the mineralization capacity of SaOs-2 osteoblasts cultured in 24- or 96-well plates, no significant difference in alizarin red S concentration could be detected regardless of the cultivation condition (Figure 6B).

## 3. Discussion

A healthy skeleton is constantly undergoing bone remodeling, resorbing damaged bone and creating new bone in a tightly regulated balance. Several chronic and debilitating bone diseases (e.g., osteoporosis) occur when the balance is disrupted, and the rate of bone resorption exceeds the rate of bone formation. Pharmacological interventions for the management of bone diseases thus primarily target and differentially modulate one of those two physiological processes [28]. However, as we continue to gain a better understanding of the complexity of bone metabolism and the key regulators involved, we may obtain further insights into the mechanisms underlying other bone-related pathologies as well and may help guide the development of new targeted therapeutics.

Bone research has primarily been performed in vivo, however, in the last decade there has been an increased interest in generating in vitro models that reduce or replicate reliance on animal testing [29]. The availability of in vitro models and a toolbox of diverse methods has since led to a detailed understanding of the biological processes that govern bone development. Although alizarin red S was discovered in 1871 [9], its staining still represents the standard method for indicating and quantifying matrix mineralization during differentiation of osteoblast cultures. However, one of its main disadvantages is its only moderate sensitivity, which hampers investigations of potentially clinically relevant substances where only small but significant effects can be expected.

The aim of this study was to optimize the alizarin red S assay readout by adding calcium chloride or calcitonin to the osteoblast differentiation medium of murine (MC3T3-E1) and human (SaOs-2) osteoblasts in order to increase mineralization. Results show that calcium chloride significantly increased the mineralization capacity of human and murine osteoblasts in a dose-dependent manner.

The stronger response to calcium chloride in human osteoblasts than in murine osteoblasts may be explained by the lack of a satisfying baseline staining in the ODM+ control. Even testing various concentrations of ascorbic acid (0.3 mM to 3 mM) and a second additional medium change per week did not result in any improvement. However, addition of calcium chloride at concentrations of 2.5 mM to the osteogenic medium led to significant mineralization and thus alizarin red S staining, emphasizing its applicability for assay optimization.

Similar effects of extracellular calcium on the mineralization of osteoblastic cells have been demonstrated previously [16,17,30]. Therefore, it is feasible that the calcium-sensing receptor senses high extracellular calcium concentrations, leading osteoblasts in vivo to sites where bone formation is required [16]. This is substantiated by the findings that high calcium concentrations not only promote chemotaxis and proliferation of MC3T3-E1-cells [13,16] but also increase proliferation and maturation of FRC cells, which are osteoblasts derived from pluripotent mesenchymal stem cells [17]. However, to the best of our knowledge, so far no one else has suggested using additional calcium chloride for better signal strength in alizarin red S staining.

In contrast to calcium, addition of calcitonin did not show any effect on mineralization compared to the acetic acid control, neither in murine nor in human osteoblastic cell lines, independent of concentration and incubation time. These results support the hypothesis that calcitonin is not directly acting on osteoblasts (neither stimulating or inhibiting) but rather indirectly via osteoclast-mediated signaling, as suggested by others [24].

Cell culture experiments are often performed in either 96- or 24-well plates, depending on availability and research question. In order to examine any possible influence of the surface area on biological mineralization, murine MC3T3-E1 and human SaOs-2 osteoblasts were cultured in parallel in both well plate formats for three weeks, with and without addition of extracellular calcium chloride. A significant difference in mineralization in MC3T3-E1 cells between 24-well and 96-well plates could only be observed in the ODM+ reference, whereas addition of calcium chloride led to a comparably strong alizarin red S staining independent of well size. No significant difference between human SaOs-2 osteoblasts cultured in 24- and 96-well plates could be detected, regardless of cultivation condition. We thus hypothesize that mineralization depends on the available surface area, but only until a certain point of cell maturation is reached. Addition of calcium chloride to the osteogenic cell culture medium most likely results in increased differentiation and maturation of osteoblasts, and thus in earlier/stronger mineralization, as detected in our experiments by alizarin red S staining. A study with human fetal osteoblastic cells (hFOBs), MC3T3-E1, and SaOs-2 cells showed that mineralization as well as mineral-to-matrix ratio increased on hydrophilic compared to hydrophobic quartz surfaces, indicating the influence of surface energy [31]. Our results suggest that not only surface energy but also surface area available for cell growth, differentiation, and mineralization can have an impact on mineralization of osteoblasts. However, future experiments are needed to verify this observation.

This study shows that mineralization of osteoblasts can be substantially enhanced by adding calcium chloride to the osteoblast differentiation medium in both murine and human osteoblastic cells in a dose-dependent manner. This approach clearly increases the sensitivity of the alizarin red S assay, making differences in the mineralization pattern of osteoblasts more visible, which is critical for studying the effects of potentially clinically relevant substances. However, it needs to be considered that calcium can interact with the substances used, which may lead to unwanted side effects.

## 4. Materials and Methods

### 4.1. Cell Lines and Growth Conditions

Depending on the experiment, the murine MC3T3-E1 (Merck Millipore, St. Louis, MO, USA) or the human SaOs-2 cell line was used. Both osteoblastic cell lines were grown at 37 °C and 5% CO_2_ in α-minimum essential medium (αMEM; Gibco, Billings, MT, USA). For mineralization, the medium was supplemented with ascorbic acid (1 mM) and ß-glycerophosphate (8 mM) (ODM+) (adjusted from Czekanska et al. [25]). The medium was changed weekly in all the experiments.

### 4.2. Experimental Setup

To optimize the alizarin red S staining of osteoblasts, murine and human osteoblast cell lines were stimulated with different concentrations of calcium chloride or calcitonin, respectively.

Human and murine osteoblasts were cultured in 96-well plates (Nalgene Nunc International, Rochester, NY, USA) in a density of 6500 cells per well. After 24 h, osteoblasts were stimulated with ODM+ medium as described above, with the exception of the negative control, where cells were cultivated with only αMEM medium (ODM−). For each concentration of calcium chloride (1 mM; 2.5 mM; 5 mM; 10 mM) or calcitonin (1 nM; 2.5 nM; 5 nM; 10 nM) added to the ODM+ medium, triplicates were used. As calcitonin was dissolved in acetic acid, an additional control with acetic acid in ODM+ medium was used. After three or four weeks (as indicated), alizarin red S staining was performed.

For the comparison of 24-well plates and 96-well plates, murine osteoblasts were incubated for only three weeks and thus at a slightly higher cell density of 50,000 (24-well plates) or 8400 cells per well (96-well plates). After 24 h, stimulation of the cells was carried out as described above. After three weeks, alizarin red S staining was performed. The alizarin red S staining consists of two different steps. First, the staining of the mineralization with the alizarin red S dye, and second the photometric quantification of the staining.

### 4.3. Alizarin Red S Staining

Staining of the mineralization with the alizarin red S dye was performed as follows: The culture medium was carefully removed and cells were washed twice with phosphate-buffered saline (PBS; Lonza, Basel, Switzerland). Cells were fixed with cold neutral buffered formalin (10%; Merck Millipore, USA) for 30 minutes (min), before formalin was removed. After washing the cells twice with distilled water (A.d.), cells were stained by adding sufficient alizarin red S staining solution (Merck Millipore, USA). After an incubation time of 30 min in the dark, the alizarin red S staining solution was removed and the cell monolayer was washed twice with A.d. The staining was analyzed under a transmitted light microscope (Leitz, Wetzlar, Germany) and pictures were taken with a Gryphax microscope camera (Jenoptik, Jena, Germany). Cells were stored at −20 °C until the photometric analysis.

### 4.4. Photometric Quantification of Alizarin Red S Concentration

The photometric analyses were performed as follows (96 well plates): 50 µL of 10% acetic acid was pipetted to the cells. Cells were detached and transferred into a 1.5 mL Eppendorf tube. Tubes were incubated for 10 min at 85 °C and shaken at 750 rpm before being transferred to ice for 5 min. After centrifugation for 10 min at maximal speed at 4 °C, 35 µL of the supernatant was transferred to a new 1.5 mL Eppendorf tube.

Then, 13 µL of ammonium hydroxide (10%; Alfa Aesar, Tewksbury, MA, USA) was added. We then transferred 40 µL of the suspension to a 96-well plate and the absorbance of solubilized calcium-bound alizarin red S was measured at 405 nm using a TECAN Spark microplate reader (TECAN, Männedorf, Switzerland). Concentrations of alizarin red S were mathematically assessed using a standard curve, which was produced as follows: 100 µL alizarin red S (40 mM) was mixed with 900 µL of a hydrochloric acid water solution (pH 4). A dilution series was built in a 1:2 relation in order to obtain different concentrations of alizarin red S (range: 31.3–200 µM), including a blank which consisted of hydrochloric acid water solution (pH 4).

The experimental procedure for the quantification of alizarin red S concentrations in 24-well plates was identical, with some modifications in the volume of the substances used: 250 µL of 10% acetic acid was added to the cells; 200 µL of the supernatant was transferred to a new 1.5 mL Eppendorf tube; 75 µL of ammonium hydroxide (10%) was added, and 50 µL of suspension was transferred to a 96-well plate for photometric measurement.

### 4.5. Statistical Analysis

All of the results are presented as the mean and standard deviation (SD) of at least three independent experiments. A Student’s *t*-test was used for testing the differences between groups. *p*-values < 0.05 were considered statistically significant.

## 5. Conclusions

Although the alizarin red S assay is considered the gold standard in quantifying osteoblast mineralization, there is still a demand for optimizations of this method in order to enhance its sensitivity. We suggest adding 2.5 to 10 mM calcium chloride to the osteogenic cell culture medium in order to enhance cell differentiation and thus bone matrix mineralization in a time- and cost-efficient manner, resulting in significantly more sensitive alizarin red S staining. Other modifications, such as addition of calcitonin or increased surface area, did not further improve the sensitivity of the alizarin red S assay.

## Data Availability

Raw data are available upon request.

## Supplementary Materials

The following supporting information can be downloaded at: https://www.mdpi.com/article/10.3390/ijms24010723/s1.
