# Supplementary material for: Optimization of the Alizarin Red S Assay by Enhancing Mineralization of Osteoblasts
Source: Int J Mol Sci. 2022 Dec 31;24(1):723. doi: 10.3390/ijms24010723 (PMC9821450; doi:10.3390/ijms24010723)
Supplement: Supplementary file 1 [file ijms-24-00723-s001.zip › ijms-2074777-supplementary.pdf]

**Table S1: Exemplary mineralization studies with or without the addition of calcium chloride and calcitonin.**

| Osteoblastic cell model | Concentration of added CaCl2 | Concentration of added calcitonin | Method(s) used for quantification of mineralization | First author and year of publication | Ref. in manuscript |
|---|---|---|---|---|---|
| MC3T3-E1 | 1.8, 2.8, 3.8 mM | - | Alizarin red S staining<br>Von Kossa staining | Yamauchi et al. 2004 | [16] |
| Fetal rat calvarial cells (FRC) | 0.5 - 2.5 mM | - | Von Kossa staining | Dvorak et al., 2004 | [17] |
| MC3T3-E1 | 1.8 – 50 mM | - | Von Kossa staining | Nakamura et al., 2010 | [30] |
| Primary mesenchymal stem cells | - | - | Alizarin Red S staining<br>Calcein Green staining | Serguienko et al., 2018 | [5] |
| Multipotent mouse mesenchymal progenitor cells (KS483) | - | - | Alizarin Red S staining<br>Staining with fluorescent dye<br>Alkaline phosphatase activity | Moester et al., 2014 | [6] |
| Calvarial cells from 6- to 8 day-old neonatal CD-1 mice | - | - | Staining with fluorescent dyes<br>Von Kossa staining<br>Alizarin Red S staining | Wang et al., 2006 | [7] |
| Human mesenchymal stem cells | - | - | Alizarin Red S staining<br>Arsenazo III calcium assay | Gregory et al., 2004 | [12] |
| Human fetal osteoblasts, MG-63, MC3T3-E1, SaOs-2 | - | - | Alizarin Red S staining<br>Von Kossa staining | Lim et al., 2008 | [31] |
| Osteoblasts isolated from the calvariae of 1-day-old newborn SD rats | - | Calcitonin-conditioned medium (3 nM) | Alizarin Red S staining | Hsiao et al., 2020 | [20] |

CaCl2, calcium chloride
